# Prediction of All-Cause Mortality Based on Stress/Rest Myocardial Perfusion Imaging (MPI) Using Deep Learning: A Comparison between Image and Frequency Spectra as Input

**DOI:** 10.3390/jpm12071105

**Published:** 2022-07-05

**Authors:** Da-Chuan Cheng, Te-Chun Hsieh, Yu-Ju Hsu, Yung-Chi Lai, Kuo-Yang Yen, Charles C. N. Wang, Chia-Hung Kao

**Affiliations:** 1 Department of Biomedical Imaging and Radiological Science, China Medical University, Taichung 404, Taiwan; dccheng@mail.cmu.edu.tw (D.-C.C.); d10119@mail.cmuh.org.tw (T.-C.H.); t10540@mail.cmuh.org.tw (K.-Y.Y.); 2 Center of Augmented Intelligence in Healthcare, China Medical University Hospital, Taichung 404, Taiwan; t35456@mail.cmuh.org.tw; 3 Department of Nuclear Medicine and PET Center, China Medical University Hospital, Taichung 404, Taiwan; d24690@mail.cmuh.org.tw; 4 Department of Bioinformatics and Medical Engineering, Asia University, Taichung 413, Taiwan; cnwang@asia.edu.tw; 5 Graduate Institute of Biomedical Sciences and School of Medicine, College of Medicine, China Medical University, No. 2, Yuh-Der Road, Taichung 404, Taiwan

**Keywords:** cardiac death prediction, CNN, ResNet-50, myocardial perfusion imaging, deep learning

## Abstract

Background: Cardiovascular management and risk stratification of patients is an important issue in clinics. Patients who have experienced an adverse cardiac event are concerned for their future and want to know the survival probability. Methods: We trained eight state-of-the-art CNN models using polar maps of myocardial perfusion imaging (MPI), gender, lung/heart ratio, and patient age for 5-year survival prediction after an adverse cardiac event based on a cohort of 862 patients who had experienced adverse cardiac events and stress/rest MPIs. The CNN model outcome is to predict a patient’s survival 5 years after a cardiac event, i.e., two classes, either yes or no. Results: The best accuracy of all the CNN prediction models was 0.70 (median value), which resulted from ResNet-50V2, using image as the input in the baseline experiment. All the CNN models had better performance after using frequency spectra as the input. The accuracy increment was about 7~9%. Conclusions: This is the first trial to use pure rest/stress MPI polar maps and limited clinical data to predict patients’ 5-year survival based on CNN models and deep learning. The study shows the feasibility of using frequency spectra rather than images, which might increase the performance of CNNs.

## 1. Introduction

Myocardial infarction and coronary artery disease are the leading causes of death of the elderly worldwide [1]. Early detection and intervention to correct the flow-limiting coronary arteries of jeopardized myocardium have shown improvements, including post-therapeutic quality of life and prevention of premature death [2]. However, correctly identifying patients who would benefit from treatment remains a challenge, which is confounded by the wide spectrum of susceptibility to myocardial ischemia and flow-limiting vasculopathy [3]. Radionuclide myocardial perfusion imaging has been applied to detect inducible myocardial ischemia. It is known to be useful for stratifying patients’ risks of imminent major adverse cardiac events (i.e., cardiac death and myocardial infarction) and determining appropriate therapeutic strategies. However, the image analysis for categorizing the findings still require multiple parametric corrections and comparisons and also depends on a representative database of healthy subjects in different populations, which complicates the process of interpreting images and prevents the generalization of the methodology to different subjects [4].

Some previous studies have used MPI for predicting the obstructive status of three coronary arteries [5] and determining if a patient has CAD (coronary artery disease) [6]. In [5], the authors used rest/stress MPI for predicting the coronary artery obstruction via a CNN and deep learning as compared with a total perfusion deficit (TPD) method. They identified 1638 patients without known coronary artery disease. Their results showed that a deep learning-based method (with a simple convolution neural network (CNN)) performed slightly better than traditional TPD measurements. The area under the ROC curve values for disease (≥70% narrowing of coronary arteries) prediction using CNN and TPD were 0.80 vs. 0.78 (per patient), respectively. In [6], the authors used relatively small images, reshaped to a size of 23 × 20, as input. Furthermore, they applied a graph convolutional neural network (GCNN) model (proposed by [7,8]) with only two convolutional layers with 64 and 128 kernels; this was a small GCNN model as compared with other CNN models for medical diagnosis. The goal was to evaluate the abilities of four different NN models (FCN, CNN, GCNN v1, and GCNN v2) for classifying a given polar map of a patient, i.e., whether it was abnormal (presence of CAD) or normal, regardless of localization. There were two types of comparison baselines: human observations and ground truth (medical findings). The NN results as compared with human observations were identified as “agreement”, whereas the NN results as compared with ground truths were identified as sensitivity and specificity. They collected 946 polar images (503 rest MPI and 443 stress MPI). Among them, the abnormal and normal ratio was nearly 1:1. They performed four-fold cross-validation. Their results showed a surprisingly high classification performance, i.e., the agreement, sensitivity, and specificity in the “rest” mode were 0.89, 0.85, and 0.93, respectively, whereas those in the “stress” mode were 0.91, 0.86, and 0.96, respectively. This was the best performance among the four NN models tested. Their contribution was to evaluate the performance of a GCNN on the classification task of myocardial event prediction, which was better than a baseline CNN.

A related study by [9] used machine learning (ML) techniques combined with clinical, stress test, imaging variables, and MPI to predict the 3-year risk of major adverse cardiac events (MACEs). A total of 2619 consecutive patients (48% men) with MPI were monitored for a MACE. Ten-fold cross-validation was used. They used 28 clinical variables, 17 stress test variables, and 25 imaging variables to train the ML models. Among them, they found “age” to be the most significant clinical variable; peak heart rate at stress, peak SBP (systolic blood pressure), and peak DBP (diastolic blood pressure) were the most significant variables in the stress tests. They did not use MPI images directly, instead, they used imaging variables. Here, a *p*-value < 0.0001 was considered to be significant. From their results, Figure 3 in [9], we note that the performance of the prediction was at specificity = 0.7 and sensitivity at about 0.76. The best score for the area under the curve (AUC) of the receiver operating characteristic curve (ROC) they reported was 0.81.

The same group published a 5-year all-cause mortality prediction study in 2017 [10]. They identified 10,030 patients at multiple centers with their coronary computed tomographic angiography. The performance of mortality prediction was measured by AUC of ROC, where the ML method showed the best performance with AUC = 0.79. We simply included their findings here, without more details, because we used image data that was different from their data.

In the mortality prediction studies, electronic health records (EHR) data have been used as materials combined with either ML or NN deep learning techniques. The following studies used EHR data from different datasets or data in-house. In [11], they included a cohort of 5436 admissions, patients diagnosed with acute myocardial infarction or post myocardial infarction syndrome, in the Medical Information Mart for Intensive Care III database (MIMIC-III) [12]. In the results, they reported using 79 variables and they found that the deep feedforward neural network (FNN) outperformed all machine learning algorithms. Some studies have used ML and EHR to predict mortality rates in a variety of other disease outcomes such as progression to type 2 diabetes [13], intensive care readmission [14], and the development of Alzheimer’s disease [15].

More recently, artificial intelligence has been implemented in many fields, including healthcare systems, and is expected to improve and reshape workflow and even our way of life. Especially in medical applications, many CNN-based studies have been published, such as in lung nodule detection [16,17], cancerous bone metastasis detection and classification on bone scintigraphy images [18,19], COVID-19 screening on X-ray images [20], and breast cancer detection on mammograms [21]. More state-of-the-art studies using CNN and deep learning in cardiovascular images can be found in [22].

In this study, we aimed to explore the feasibility of using artificial intelligence to assisting in classifying the risk of 5-year all-cause mortality in patients who had recently experienced an adverse cardiac event by direct analysis of basic acquisition of myocardial perfusion imaging without the need to compare the predefined normal database from the healthy subjects or to acquire additional advanced images with specific processing module (e.g., electrocardiogram gated images with compatible processing software). The results might have the potential to create a new pathway of personalized assessment that could contribute to establishing tailor-made healthcare plans.

Our innovation, in this study, is using the frequency spectra of bull’s-eye images as the input of CNN model, instead of using raw images. This inspiration comes from GCNN [7,8]. More details are given in the Discussion Section.

## 2. Materials and Methods

### 2.1. Materials

From November 2007 to October 2018, a total of 1162 consecutive patients were referred for thallous-201 chloride (Tl-201) stress/redistribution myocardial perfusion single photon emission computed tomography (SPECT). Two hundred and ninety-two patients were excluded from further analysis, including 7 pediatric subjects (unlikely underlying etiologies of atherosclerotic coronary disease), 18 patients with inappropriate image qualities, and 267 patients lost for further clinical follow-up. Of the remaining 870 patients, 577 were dead in 5 years after myocardial perfusion SPECT and the remaining 293 patients were alive, according to the death data of the clinical research database of the China Medical University Hospital. The pharmacologic cardiac stress method was used with dipyridamole administered at 0.56 mg/kg intravenously over a 4-min period. Tl-201 was injected with 2.5 millicuries (mCi), 3 to 5 min after the completion of dipyridamole infusion. Stress imaging started 10 min after completion of dipyridamole infusion and redistribution images were acquired 4 h later. The SPECT acquisition was performed with a Millennium MG dual-head gamma camera, Infinia/Hawkeye 4 SPECT/CT, or Discovery NM/CT 670 SPECT/CT (GE Healthcare, Waukesha, WI, USA) equipped with low energy or extended low energy general purpose collimators. The SPECT images were acquired with 32 projections, 40 s per projection, and 180° arc (from 45° right anterior oblique to 45° left posterior oblique) and stored in a 64 × 64 matrix. For further interpretation, three-orthogonal sections along the short, horizontal, and vertical long axes of the left ventricular images were reconstructed, and polar maps of the short axial images were also created with the vendor-provided Xeleris Workstation (GE Healthcare, Waukesha, WI, USA). This study was approved by the Institutional Review Board (IRB) of the China Medical University and Hospital Research Ethics Committee (DMR99-IRB-(CR-9)).

The collected MPI images were in DICOM format, only age and gender information were preserved, and all private connections were removed. The spatial resolution of the raw images was 1024 × 512 pixels. However, only bull’s-eye ROIs were extracted for follow-up preprocess, and the heart/lung ratio was extracted. The heart/lung ratio was computed by selecting two ROIs from the heart and lung regions. This process was performed manually by a physician (co-author Y.C. Lai) and confirmed by two other physicians (co-authors C.H. Kao and T.C. Hsieh). The remaining parts of an image were ignored. The 862 patients were aged between 28.2 and 99.6 years, with an average age of 69.2 ± 12.3 years; there were 487 males. These 862 cases were used to perform Experiment 1, i.e., the baseline. The lung/heart ratios were: (min, max, and mean ± std) = (0.2, 1, and 0.39 ± 0.11) for stress and (0.21, 1, and 0.41 ± 0.09) for rest. Since the outcome was death or not in 5 years after an adverse cardiac event, this was a retrospective study and traced by patient records.

**Figure 1:**
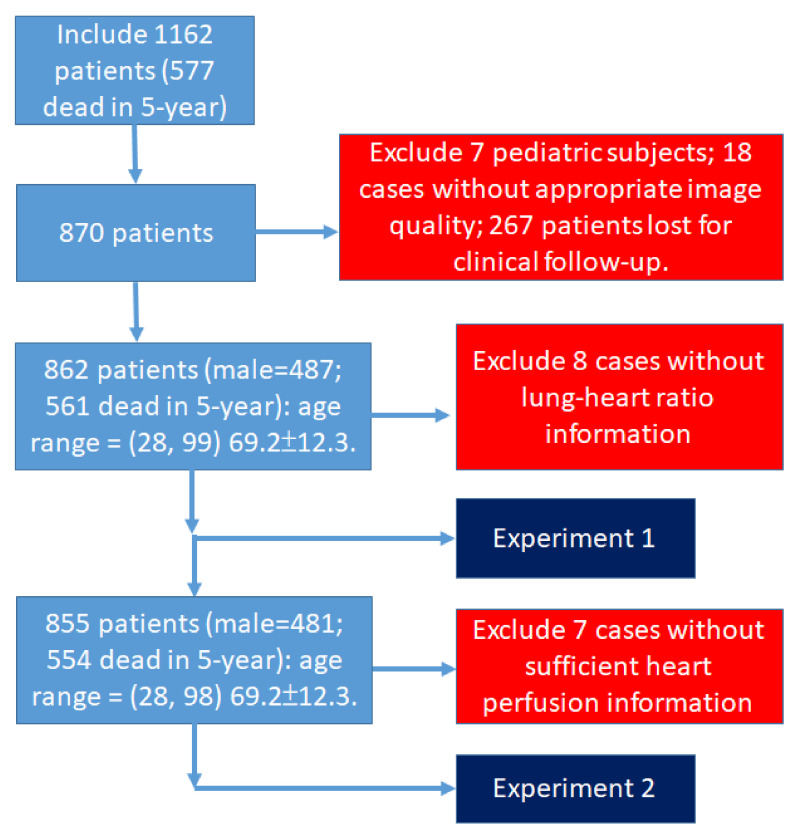
The patient data structure and flowchart.

We noted that 7 cases showed poor perfusion quality; therefore, these 7 cases were removed, and the remaining 855 patients were used to perform Experiment 2, i.e., our novelty finding. The patient data structure is shown in Figure 1.

### 2.2. Methods

#### 2.2.1. Image Preprocessing

The bull’s-eye region was extracted by a circle detection algorithm, i.e., Hough circle transform [23] using python package function %cv2.HoughCircles%. Since the raw images were saved by different radiographers, the image resolution could be slightly different. We simply chose the radius of the majority as a standard radius. All other sizes were rescaled to fit this standard. After the circle detections, all bull’s-eye regions were extracted and fitted to an image of size 220 × 440. We referred to this image as a “raw bull’s-eye” image. There were 862 “raw bull’s-eye” images.

**Figure 2:**
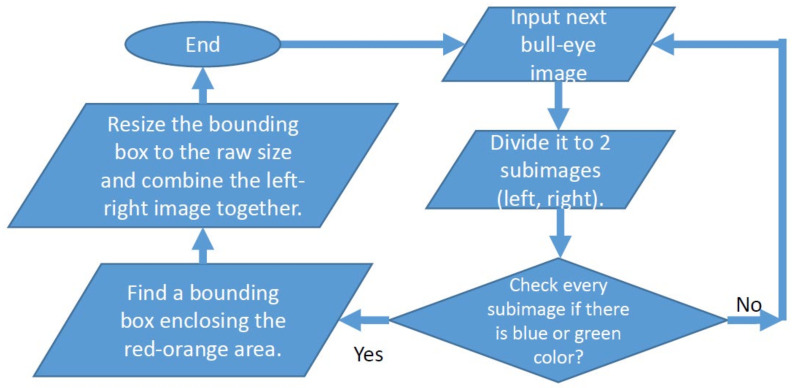
The image preprocess algorithm. The purpose of this algorithm is to exclude the region outside the heart, and therefore the bull’s-eye image contains only heart perfusion information. It is a type of noise-removal technique. The resulting “pure bull’s-eye” images were used in Experiment 2.

The raw bull’s-eye images needed to contain pure heart perfusion information. However, due to the skill variations of different radiographers, the “raw bull’s-eye” images could contain information outside the heart area. This was not expected, and therefore we developed an algorithm to fix the problem, as shown in Figure 2. After this process, the bull’s-eye images contained pure heart perfusion information without other noises, and we referred to these as “pure bull’s-eye” images. These 855 “pure bull-eye” images were used in Experiment 2.

In Experiment 2, we applied Fourier transform to extract the frequency spectra from the “pure bull-eye” images. The zero frequency was removed, and the remaining frequencies were preserved to test different CNN models.

#### 2.2.2. Convolutional Neural Networks (CNNs)

There have been many CNN models developed in recent years, for example, ResNet50V2 [24,25,26], ResNet101V2 [24,25,26,27], MobileNetV1 [28,29], MobilNetV2 [28,30], Xception, VGG16, EfficientNetB0, and DenseNet169. These are current state-of-the-art CNN models. A CNN model can extract image features fully automatically from the training data (images and clinical data) and perform a classification task in a network. In our neural network, we input: (1) bull’s-eye image, (2) lung/heart ratio, (3) patient’s age, and (4) patient’s gender; the output was: survival in 5 years, either yes or no. The image and clinical data were combined through a “concatenation” technique, using the python package function %keras.layers.concatenate%. The age was normalized by dividing by 100, the gender used one-hot encoding, and the lung/heart ratio was directly used after normalization.

#### 2.2.3. Frequency as Input

In order to explore the difference between using raw color images and their frequency spectra, we applied fast Fourier transform (FFT, used %numpy.fft.fft2%) on the “pure bull’s-eye” images. Notably, the zero frequency was deleted after the FFT and the remaining parts were fed into the CNN models. The real and imaginary parts were combined by using the package absolute function %numpy.abs%. The three channels (red, green, and blue) were separately computed. The Log operation was applied to enhance the weights of the high frequency component by using the %numpy.log10% function.

## 3. Results

### 3.1. Image Preprocessing

**Figure 3:**
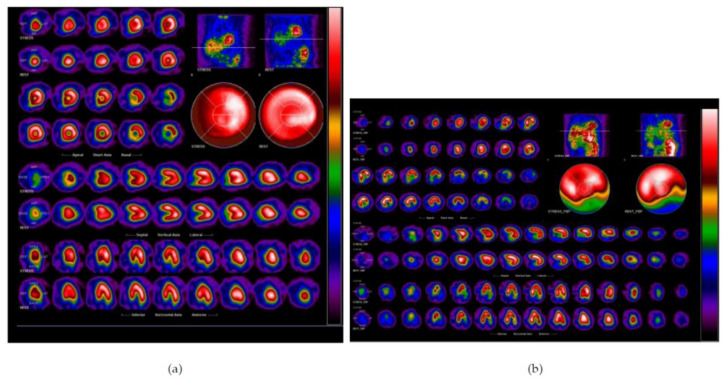
Raw MPI images. The heart perfusion information was correctly captured by the “bull’s-eye” image in (**a**). However, the heart perfusion information was not put in the center of the “bull’s-eye” in (**b**). This is an example of the difficulty encountered in our study.

The raw MPI images were in different sizes and appearances. Figure 3 illustrates two MPI images as examples with different image sizes. Moreover, we noted that the heart perfusion information in Figure 3a is correctly captured by the “bull’s-eye” image, since there is no blue and green color surrounding it, as can be seen in Figure 3b. The red and orange colors denote good blood perfusion, while the blue and green colors denote ischemia. These differences might be caused by the skills of different radiographers. An experienced radiographer is able to achieve good image quality. This is a prospective study, and therefore we were unable to repeat the image capturing process.

**Figure 4:**
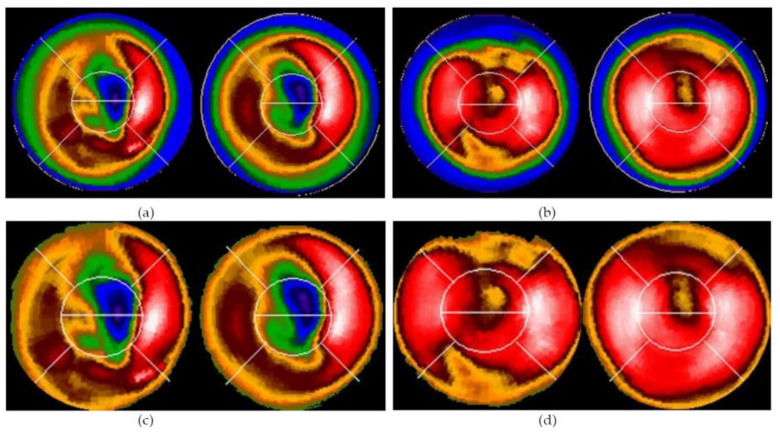
The extracted “bull’s-eye”: (**a**,**b**) “raw bull’s-eye” and (**c**,**d**) “pure bull’s-eye” after processed by our algorithm (in Figure 2). (**a**,**c**) are Case 2, (**b**,**d**) are Case 47.

Figure 4 shows examples of the extracted “raw bull’s-eye” and “pure bull’s-eye” images.

### 3.2. Results of CNNs

In total, eight state-of-the-art CNN models were tested. Table 1 (Experiment 1) shows the accuracy values for prediction performance. Each value was the median of three tests. In each test, 10% of the data were randomly chosen to be test data, 90% of the data were training data. The final median value is shown in the bottom row of Table 1. Each CNN model was tested 13 × 3 = 39 times. This was an ablation study. From the results, we found that the best CNN model was ResNet, which had a median accuracy of 0.70, i.e., the baseline result. The best accuracy for each CNN model is marked in bold.

In order to explore the impact of using the frequency spectra instead of normal images as input, we designed Experiment 2 (see Table 2). In Experiment 2, seven poor quality cases were removed. In total, 855 cases were tested. Similarly, 10% of the data were randomly chosen as test data and the remaining 90% of the data were used as training data. Surprisingly, all CNN models had better performance and the increments were about 7~9%. This was a novel finding.

We repeated the tests five times using the ResNet50V2 model with epoch = 80, batchsize = 32, frequency spectra as input, and the confusion matrices are shown in Table 3. From the results, we observe that the model is stable and has a better performance than using raw image as input.

Since three clinical parameters were used, we designed an experiment in order to explore which parameters were significant. Each clinical parameter was removed, and the experiments were repeated three times using the ResNet50V2 model. The results are shown in Table 4. From the results, we observe that the parameters age and gender are more significant than lung/heart ratio.

## 4. Discussion

In this study, our contributions are two-fold: (1) This is the first trial to use MPI information and as few as possible clinical data as input for eight state-of-the-art CNN models for 5-year survival predictions in patients after experiencing an adverse cardiac event. (2) This is the first trial to use the frequency spectra of images as input for a CNN and find a better performance. This is a novel finding, which could motivate future researchers to consider different types of input rather than only raw images. We emphasize that not all images were suitable for using frequency spectra as the input. The CNN can better predict 5-year survival rate, while this is a difficult task for humans. This might be a consideration in deciding if we need to transfer images to frequency spectra and use it as input. The reason that using spectrum as input outperform raw images as input might be owing to: We human beings are well-trained to recognize natural and man-made objects by extracting shapes, colors, and texture features. However, we are not well-trained on recognize objects in frequency domain. The goal of this study is to predict the risk of 5-year all-cause mortality of patients experienced an adverse cardiac event. This is a linkage between MPI and mortality. The spatial information might have less information than the spectrum. We note that it is very hard for physicians to do the prediction. In other words, humans cannot extract enough features efficiently from MPI to predict mortality.

Many studies have compared their results with previous studies; however, there are limited related studies for a comparison with our study. In addition, a fair comparison should be based on the same dataset, in this case, we did not find an open dataset of MPI as a benchmark. Therefore, we compared eight state-of-the-art CNN models and we conducted an ablation study by changing combinations of different epochs and batch sizes. To obtain reliable accuracy, we reported the median value of many test trials instead of the best results that might have been due to luck.

**Figure 5:**
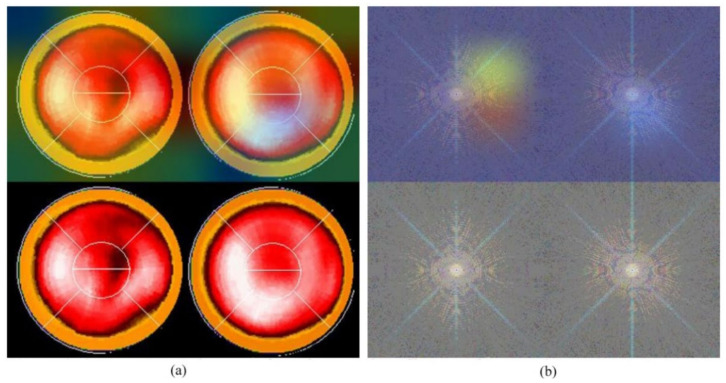
The GradCAM plots, Case 60: (**a**) Results of Experiment 1, the lower image is the “pure bull’s-eye” image, and the upper image is its GradCAM visualization; (**b**) results of Experiment 2, the lower image is the frequency spectrum computed from (**a**), the pure bull’s-eye image, and the upper image is its GradCAM visualization.

**Figure 6:**
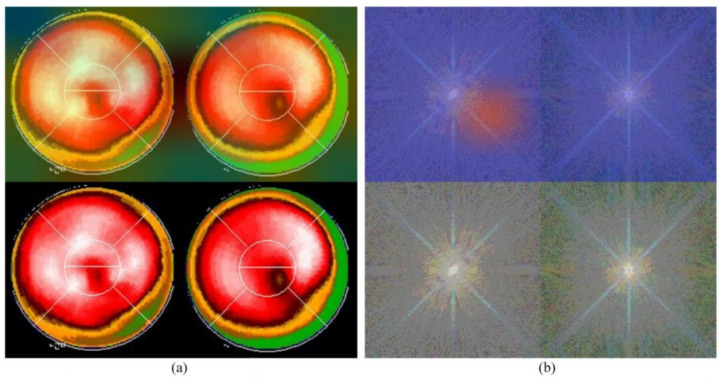
The GradCAM plots, Case 170: (**a**) Results of Experiment 1, the lower part is the “pure bull’s-eye” image, and the upper part is its GradCAM visualization; (**b**) results of Experiment 2, the lower part is the frequency spectrum computed from (**a**), the pure bull-eye image, and the upper part is its GradCAM visualization.

**Figure 7:**
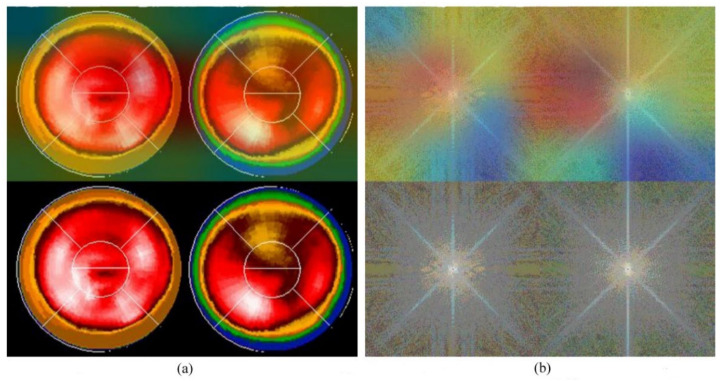
The GradCAM plots, Case 595: (**a**) Results of Experiment 1: the lower part is the “pure bull-eye” image, and the upper part is its GradCAM visualization; (**b**) results of Experiment 2: the lower part is the frequency spectrum computed from (**a**), the pure bull’s-eye image, and the upper part is its GradCAM visualization.

**Figure 8:**
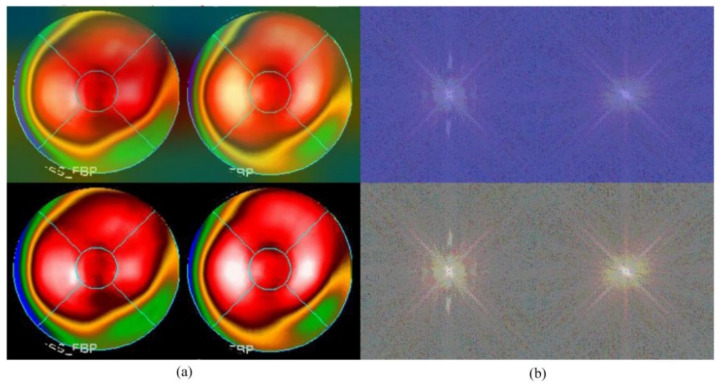
The GradCAM plots, Case 674: (**a**) Results of Experiment 1: the lower part is the “pure bull’s-eye” image, and the upper part is its GradCAM visualization; (**b**) results of Experiment 2: the lower part is the frequency spectrum computed from (**a**), the pure bull’s-eye image, and the upper part is its GradCAM visualization.

Neural network-related methods are often challenged by questioning the mechanism of computation, especially as to how an NN knows the result and from where an NN could make such a decision. This is the so-called “black-box” question. An interesting study, in 2019 [31], provided a technique namely gradient-weighted class activation mapping (Grad-CAM), which provided an intuitive visualization of where the NN was focusing. We call the region that the NN focuses on the “hot zone” (or heat map in python program). The hot zones actually are the weights in NN, if the weights have larger values, then, the region has a greater influence on making a decision. The hot zone is performed by color superimposed on the raw image, a hot color representing a greater influence. We used this technique and provided an intuitive visualization where the CNN was focusing. Figure 5 and Figure 6 are examples: Figure 5a and Figure 6a are Experiment 1 and Figure 5b and Figure 6b are Experiment 2. The lower images in the figures are raw images (pure bull’s-eye and its FFT), and the upper images are their GradCAM visualization. From the hot zones, we can observe that a hot zone is hard to observe since the raw image also has no hot color in the bull’s-eye. However, in Experiment 2, we can easily observe that the CNN only focused on the left bull’s-eye, the stress phase of MPI. This is an interesting finding, which means that the CNN makes a decision only on the stress phase of the bull’s-eye. Most results are similar to Figure 5 and Figure 6; however, we observed some rare cases, as shown in Figure 7 and Figure 8. In Figure 7, we note that the CNN makes a decision based on two bull’s-eyes, especially on the right bull’s eye (the rest phase). In Figure 8, the CNN makes a correct decision, but we do not see any hot region in the heat map. In Case 674, we tend to believe that the CNN uses guessing, since the heart was not well settled in the center of the bull’s-eye. An interesting question is, “What is the percentage of guessing in correct and incorrect classifications?” We found that 18% and 30% guessing existed in correct and incorrect classifications, respectively. This makes sense, since guessing has a lower rate of correctness.

The accuracies of 5-year survival predictions reached 0.77 for most of the CNN models. The limitations of this study may be due to the following reasons: (1) Our MPI images were obtained by different radiographers, who had different experiences. Some MPI images had poor quality in perfusion information, i.e., the heart perfusion information was not correctly centered in the image. (2) We used only gender, lung/heart ratio, and patient age as input clinical data, which is very limited. According to a previous study by [9], peak heart rate at stress, peak SBP (systolic blood pressure), and peak DBP (diastolic blood pressure) are significant for predicting adverse cardiac events. In addition, some simple clinical data, such as body mass index (BMI), should be considered, which did not exist in our dataset.

## 5. Conclusions

In this study, we developed CNN-based models using MPI and data of three clinical parameters to predict the survival of patients who had experienced an adverse cardiac event. The accuracy of predictions reached 0.77 for six state-of-the-art CNN models when the input of CNN was frequency spectra using fast Fourier transform on images. Our findings could provide direction for future studies to consider different types of input for a CNN, instead of using the traditional method of raw images.

## Figures and Tables

**Table 1:** Experiment 1, the baseline. Eight state-of-the-art CNN models were tested. Images were input. The values in the table show accuracy. Each value was the median of three tests with different initializations (10% of the data were test data, 90% data were training data, total 862 patients).

Epoch	Batchsize	ResNet 50V2	ResNet 101V2	Mobile NetV1	Mobile NetV2	Xception	VGG16	EfficientNetB0	DenseNet169
40	16	0.71	0.71	0.67	0.61	0.68	0.62	0.64	0.70
60	16	0.71	0.67	0.70	0.67	0.63	0.68	0.72	0.66
60	32	0.69	0.68	0.67	0.68	**0.71**	0.64	0.69	**0.71**
60	64	0.64	0.71	0.72	0.69	0.70	0.67	**0.76**	0.67
80	16	0.70	0.71	0.69	0.69	0.68	**0.72**	0.71	**0.71**
80	32	0.70	0.68	0.64	0.69	0.68	0.67	0.68	0.69
80	64	0.66	0.69	0.72	0.63	0.70	0.70	0.71	**0.71**
120	16	0.70	0.70	0.69	**0.74**	0.66	0.70	0.64	0.67
120	32	0.71	0.72	0.64	0.72	0.64	0.68	0.69	0.69
120	64	0.61	0.70	**0.74**	0.70	0.63	0.62	0.68	0.66
160	16	0.69	0.66	0.70	0.68	0.68	0.69	0.70	0.66
160	32	0.68	**0.74**	0.68	0.67	0.67	0.67	0.68	0.66
160	64	**0.75**	0.69	0.63	0.70	0.68	0.70	0.67	0.68
Median		0.70	0.70	0.69	0.69	0.68	0.68	0.69	0.68

**Table 2:** Experiment 2. Eight state-of-the-art CNN models were tested. The frequency spectra were input. The value in the table was accuracy. Each value was the median of three tests with different initializations (10% of the data were test data, 90% of the data were training data, total 855 patients).

Epoch	Batchsize	ResNet 50V2	ResNet 101V2	Mobile NetV1	Mobile NetV2	Xception	VGG16	EfficientNetB0	DenseNet169
40	16	0.76	0.77	0.77	0.76	0.71	0.77	0.77	0.77
80	32	0.78	0.77	0.70	0.76	0.76	0.76	0.77	0.77
160	64	0.77	0.76	0.77	0.71	0.76	0.77	0.76	0.76
Median		0.77	0.77	0.77	0.76	0.76	0.77	0.77	0.77

**Table 3:** Experiment 2. ResNet50V2 model was tested 5 times with different shuffles, 10% data were test data, 90% data were training data. The confusion matrices are listed. Total of 855 patients.

Number		1		2		3		4		5	
Prediction		Death	Alive	Death	Alive	Death	Alive	Death	Alive	Death	Alive
Ground truth	Death	54	6	50	10	56	8	54	6	54	6
	Alive	11	15	13	13	12	10	13	13	11	15
Accuracy		0.80		0.73		0.77		0.78		0.80	

**Table 4:** The significance of clinical parameters.

Parameter Absent		Lung/Heart Ratio	Age	Gender
Number	1	0.79	0.58	0.62
	2	0.77	0.62	0.70
	3	0.71	0.60	0.70

## Data Availability

Not applicable.
